# Comparison of two optimization algorithms (VOLO^TM^, SEQU) for CyberKnife® treatment of acoustic neuromas, lung metastases, and liver metastases

**DOI:** 10.1002/acm2.14144

**Published:** 2023-09-06

**Authors:** Martin Thiele, Kirsten Galonske, Iris Ernst

**Affiliations:** ^1^ German CyberKnife‐Center Soest Germany; ^2^ German Center for Stereotaxy and Precision Irradiation Soest Germany

**Keywords:** CyberKnife®, SRS, SBRT, optimization algorithm

## Abstract

**Introduction:**

Two optimization algorithms VOLO™ and sequential optimization algorithm (SEQU) are compared in the Precision® treatment planning system from Accuray® for stereotactic radiosurgery and stereotactic body radiotherapy (SBRT) treatment plans. The aim is to compare the two algorithms to assess if VOLO™ is better of SEQU in certain treatment site.

**Materials and Methods:**

Sixty clinical treatment cases were compared. Entities include Acoustic neuroma (AN), lung metastases, and liver metastases. In each entity, 10 SEQU and 10 VOLO™ treatment plans were optimized. The Ray‐Tracing calculation algorithm was used for all treatment plans and the treatments were planned exclusively with fixed cones (5–50 mm). The number of nodes, beams, total MU, and treatment time were compared. Conformity index (CI), new conformity index (nCI), homogeneity index (HI), gradient index (GI), and target coverage were examined for agreement. D_min_, D_mean_, D_max_, D100%, D98%, and D2% dose in the target volume as well as exposure to organs at risk was checked. To determine peripheral doses, the isodose volumes from V10% to V98% were evaluated.

**Results:**

AN treatment plans showed significant differences for the number of nodes, beams, total MU, treatment time, D98%, D100% for the target volume, and the doses for all organs at risk. VOLO™ achieved better results on average. Total MU, treatment time, coverage, and D98% are significantly better for VOLO™ for lung metastases. For liver metastases, a significant reduction in number of nodes, total MU, and treatment time was observed for VOLO™ plans. The mean target coverage increased slightly with VOLO™, while the mean CI deteriorated slightly. The averages of D_min_, D_mean_, D98%, D100%, and V80% resulted in a significant increase for VOLO™.

**Conclusion:**

The results of the present study indicate that VOLO™ should be used in place of SEQU as a standard for AN cases moving forward. Despite the lack of significance in the lung and liver cases, VOLO™ optimization is recommended because OAR sparing was similar, but coverage, D_min_, and D_mean_ were increased, and thus better tumor control can be expected.

## 1 INTRODUCTION

The CyberKnife®, which was developed by John Adler,^1^ is a dedicated robotic system for carrying out stereotactic radiosurgery (SRS) and stereotactic body radiotherapy (SBRT).^2^ Beams can be collimated using the IRIS™‐aperture,^3^ fixed cones, or InCise™ multileaf collimator (MLC).^4^ Optimization algorithms are used during the treatment planning process in radiotherapy to enclose the target volume with the prescribed dose while sparing the surrounding organs at risk (OARs) optimally. The underlying mathematical models to solve problems of that kind can be computationally intensive.^5^

By performing the dose calculation on the graphic processing unit (GPU) instead of the central processing unit (CPU), calculation time can be minimized. The first optimization algorithm which used GPU‐based dose calculation for Tomotherapy® treatment planning was implemented as early as 2012.^6^ The new treatment planning system (TPS) Precision™ from Accuray® (Accuray Inc., Sunnyvale, CA, USA) has introduced a GPU‐based Volume‐Optimization algorithm called VOLO™. CyberKnife® users can now use this instead of the already available sequential optimization algorithm (SEQU).^7^ The VOLO™ algorithm is characterized by significantly shorter treatment times (19%) and optimization times (on average, a factor of 10 shorter).^8^

SEQU is a powerful approach used to solve complex optimization problems. It involves the step‐by‐step improvement of a solution by adjusting its variables in a sequential manner. The user specifies the sequence, and there are no weighting factors. Thus, a single optimization step contains a single cost function that corresponds to either an absolute or a loose limit value. With the VOLO™ algorithm, the absolute constraints or clinical goals specified by the user are weighted and combined into a single cost function. This means that several individual criteria cost functions, as with SEQU, are replaced by a single multi‐criteria cost function.^9^ Up to five clinical goals can be set for each volume.

To understand SEQU and the VOLO™ algorithm in more detail, we can refer to the works of Calusi et al., Schüler et al., and Zeverino et al.^8^, ^9^, ^10^ These researchers have conducted extensive studies on this topic and have provided valuable insights into the theoretical foundations and practical applications. Their plan comparisons regarding both optimization algorithms focused on IRIS™‐aperture and MLC collimator systems.

In this paper, fixed cone‐based plans were selected. Sixty clinical CyberKnife® treatment plans have been optimized with both algorithms and compared dosimetrically to find out which is the better optimization algorithm for treatment planning. Our three main entities: acoustic neuromas (AN), lung metastases, and liver metastases, are examined. The peripheral doses of both optimization algorithms are highlighted.

## 2 MATERIAL AND METHODS

### 2.1 Optimization algorithms

The TPS Precision® (Version 2.0.1.1, Accuray Inc., Sunnyvale, CA, USA) offers the user two optimization algorithms: SEQU and VOLO™.

The SEQU algorithm executes the absolute constraints specified by the user, such as maximum target coverage with the prescribed isodose, homogeneity of dose in the target volume, conformity of dose distribution around the target volume, maximum or average dose to OARs, and reduction of total monitor units (MU) step by step. There is also the function of beam reduction, time reduction, node reduction, and segment reduction (the latter only for MLC). With beam reduction, the user specifies a MU cutoff value for all beams. All beams that do not comply with this value are deleted. The treatment plan is re‐optimized for the remaining beams. That leads to a reduction in treatment time, in total MU, and a possible deterioration in the dose distribution.

The basic idea of the VOLO™ algorithm comes from intensity‐modulated radiation therapy (IMRT), where a fluence map is optimized in the first step and is sequenced as a set of segments in the second step. The weighting of the individual segments can optionally be optimized. It traditionally applies to beams defined by a multi‐leaf collimator.^11^ With few modifications, the algorithm can be applied to the CyberKnife® VSI™ system using an IRIS™‐aperture or 12 fixed cones (circular field sizes for these two types of collimators are from 5 to 60 mm). The user can set the maximum number of nodes and the maximum and minimum target volume dose. Maximum dose values and volume‐dependent doses can be specified for OARs. Parameters such as the maximum number of beams, and maximum or minimum MU per beam per fraction can also be adjusted. It is possible to define up to 500 optimization iterations.

### 2.2 Cases selection and treatment planning

Sixty previously applied treatment plans with single lesions were chosen. The entities include 20 AN, 20 lung metastases, and 20 liver metastases. Ten SEQU plans and 10 VOLO™ plans per entity were selected. The applied plans, which were created from one dosimetrist, were optimized with the other algorithm and compared dosimetrically (the total compared plans are 120). The prescription dose, collimator type, collimator size, optimization shells, and blocked structures were retained during the re‐planning. The Ray‐Tracing calculation algorithm^12^ was used for all treatment plans and the treatments were planned exclusively with two fixed cones (5–50 mm). Beam reduction was used with a value of 10 MU per beam. In our daily practice, no time reduction or node reduction was used. Patients with AN were tracked with 6D Skull tracking (Accuracy is 0.5 mm^13^), and patients with lung metastases or liver metastases got Xsight® Spine tracking (Accuracy is 0.49 mm ± 0.22 mm^14^).

Table 1 shows PTV size, fraction, total dose, and median isodose % for the three studied tumor entities. Treatment planning is performed with the constraints listed in Table 2. For AN cases, the PTV results from GTV + 1 mm, and for lung metastases and liver metastases, the PTV is obtained by adding a margin of 1 mm to the ITV. The goal is to have 98% of the PTV enclosed with the prescribed isodose.

**TABLE 1:** Overview of three tumor entities with median PTV size, prescription dose and prescription isodose.

Tumor entity	Median PTV Size cm^3^ (range)	Prescription	Median Isodose % (range)
		Fraction/total dose in Gy	
AN	1.02 (0.11‐4.71)	1/13.0	80 (70‐80)
Lung metastases	11.3 (1.77‐73.73)	3/37.5	65 (65)
Liver metastases	27.24 (5.5‐142.45)	3/37.5	65 (63‐75)

**TABLE 2:** Applicable dose constraints at the German CyberKnife‐Center for different OARs and fraction schemes.

Organ at risk	Fx	Vol cm^3^ or %	Volume Max (Gy)	Max Point Dose (Gy)	Mean Dose (Gy)	Endpoint (≥Grade 3)	Reference
Brainstem	1	<1	10	15		Cranial neuropathy	^15^
Optic pathway	1	<0.2	8	10		Neuritis	^15^
Optic nerve	1			12			^16^
Eye	1			5			^17^
Lens	1			2			^18^
Spinal cord	3			16		Myelitis	^19^
	3	<0.25	18	22			^15^
	3	<1.2	11.1				^15^
Lung, bilateral (without PTV)	3	20 %	20				^17^
	3	30 %	10				^17^
	3	50 %	5				^17^
Heart	3	<15	24	30		Pericarditis	^15^
Esophagus	3	<5	17.7	25.2		Stenosis/fistula	^20^
	3	<5	21	27			^15^
	3	2	35				^19^
	3	2	35				^19^
Liver	3	700^a^	17.1^b^			Basic liver function	^15^
	3	33 %	21				^21^
	3	30 %	24				^19^
	3	50 %	18				^19^
Kidney	3				6		^19^
Stomach	3	<10	21	24			^15^
	3	2	30				^19^

^a^ Critical Volume Dose.

^b^ Crit. Vol. Dose Max.

### 2.3 Comparison parameters

The number of nodes, the number of beams, the total MU, and the treatment time were compared. The treatment time includes the time of CyberKnife®‐movement from node to node and the time of taking x‐ray images for tracking. The radiotherapist can change the number of verification exposures during the treatment. The frequency of the x‐ray image repetitions can be set between 5 and 150 s and is set to 90 s for intracranial lesions and to 150 s for extracranial lesions.

Conformity index (CI), new conformity index (nCI), homogeneity index (HI), and target coverage were examined for agreement.

$$CI\;=\frac{PIV}{TIV}$$

CI is calculated from the ratio of the volume of the prescribed isodose (PIV) to the tumor volume that the prescribed isodose covers (TIV).^22^ It is the reciprocal value for healthy tissue proposed by Lomax et al.^23^

$$nCI\;=\frac{PIV\;\cdot \;TV}{{\left(TIV\right)}^{2}}$$

The nCI assesses healthy tissue and the quality of tumor coverage. The target volume (TV) is the volume of the planning target volume (PTV). Both CI and nCI are 1.0 if PIV = TV = TIV. Paddick´s conformity index (PCI) is the reciprocal of the nCI.^24^

$$HI\;=\frac{D_{max}}{D_{pres}}$$

The homogeneity index (HI) is defined by the ratio of the D_max_ and the prescribed dose (D_pres_) and it describes the homogeneity of the dose distribution in the target volume.^25^

$$GI\;=\frac{V_{\frac{pres}{2}}}{V_{pres}}$$

To determine the dose falloff outside the target, we used the dose gradient index (GI). It is defined as the ratio of the volume of half the prescription isodose to the volume of the prescription isodose.^24^

$$Coverage\;in\;\%\;=\frac{TIV}{TV}\;\cdot 100$$

The coverage is the volume of the target receiving equal to or greater than the prescribed dose (TIV) divided by the total volume of the target (TV) multiplied by 100.

The target volumes were compared using the minimum dose (D_min_), mean dose (D_mean_), and maximum dose (D_max_). A comparison of the maximum dose exposure of the organs at risk listed in Table 2, and the volume limit V21 < 700 cm^3^ for the liver was performed. The D100%, D98%, and D2% dose in the target volume of the two optimization algorithms were also compared. To determine peripheral doses, the volumes of the V10%, V20%, V40%, V60%, V80%, and V98% isodoses were evaluated.

The results of the comparison between SEQU and VOLO™ were statistically evaluated with OriginPro® 2016 (Version 9.3.266, OriginLab Corporation, Northampton, MA, USA). The Shapiro‐Wilk test (significance level α = 0.05) was used to check whether the data were distributed normally. For normally distributed data, a paired sample *t*‐test was carried out (significance level α = 0.05) and for non‐normally distributed data, an evaluation was carried out with the Wilcoxon signed‐rank test for paired samples (significance level α = 0.05).

### 2.4 Clinical evaluation

Clinical evaluation of the treatment plans was performed by our experienced team of physicians. In a blinded plan evaluation, physicians were asked to decide which of the two treatment plans to choose. The evaluation criteria of coverage, conformity, exposure of organs at risk, peripheral dose, and treatment time were rated using a scale. The scale ranged from –2 to +2, with –2 representing a substantial improvement in SEQU optimization and +2 representing a substantial improvement in the VOLO™ plan; 0 represents the equivalence of the two treatment plans.

## 3 RESULTS

### 3.1 Plan parameters of the entire study

**FIGURE 1:**
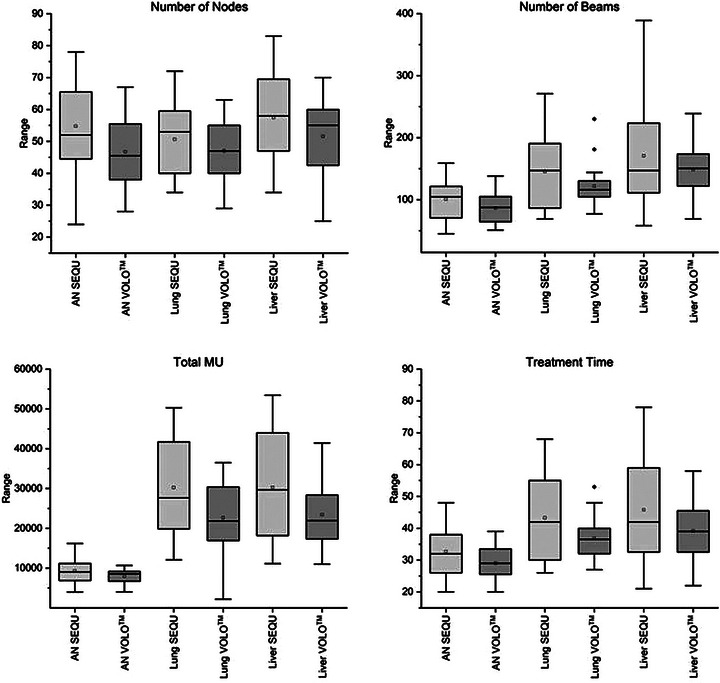
Number of nodes, number of beams, total MU and treatment time for AN, lung and liver metastases for both optimization algorithms.

Tables 3, 4, 5 present all examined parameters for each entity. The parameters marked with (*) were found to be normally distributed using the Shapiro‐Wilk test. These parameters include the number of nodes, total MU, and treatment time for the three entities studied. Plan Optimization with VOLO™ decreased the number of nodes by 14% for AN, by 8% for lung metastases, and by 9% for liver metastases. Total MU is significantly different with a *p*‐value of ≤0.05 for AN, lung, and liver treatment. Total MU decreased by 14% (1313 MU) for AN treatment, 25% (7635 MU) for lung treatment, and 23% (6896 MU) for liver treatment by using VOLO™ optimization instead of SEQU. VOLO™ optimized plans also reduced treatment time by an average of 12% in AN treatment, 14% in lung treatment, and 15% in liver treatment. Figure 1 displays the box plots of the number of nodes, number of beams, total MU, and treatment time. VOLO™ optimized plans result in higher CI and nCI on average in the examined regions. V98% to V20% are also higher or the same when using VOLO™ optimization. In 50 out of 60 plan comparisons, a higher coverage is obtained with VOLO™.

**TABLE 3:** Means and standard deviations of all parameters for AN treatment.

	Parameter	VOLO™	SEQU	*p*‐value
**AN**	**Number of nodes**	**47 ± 11**	**55 ± 15**	**≤0.05** ^a^
	**Number of beams**	**86 ± 26**	**101 ± 34**	**≤0.05** ^a^
	**Total MU**	**7955 ± 1710**	**9268 ± 3283**	**≤0.05** ^a^
	**Treatment time (min)**	**29 ± 5**	**33 ±8**	**≤0.05** ^a^
	CI	1.31 ± 0.27	1.28 ± 0.28	n. s.
	nCI	1.31 ± 0.27	1.29 ± 0.28	n. s.
	HI	1.27 ± 0.06	1.26 ± 0.04	n. s.
	GI	4.87 ± 0.75	4.74 ± 0.67	n. s.^a^
	**Coverage (%)**	**99.7 ± 0.5**	**99.0 ± 0.8**	**≤0.05**
	D_min_ (Gy)	12.88 ± 0.67	12.67 ± 0.61	n. s.
	D_mean_ (Gy)	14.82 ± 0.70	14.84 ± 0.46	n. s.
	D_max_ (Gy)	16.50 ± 0.71	16.50 ± 0.71	n. s.
	**D100% (Gy)**	**13.05 ± 1.01**	**12.67 ± 0.61**	**≤0.05**
	**D98% (Gy)**	**13.45 ± 0.44**	**13.15 ± 0.33**	**≤0.05**
	D2% (Gy)	16.16 ± 1.07	16.36 ± 0.70	n. s.
	**Brainstem D_max_ (Gy)**	**7.23 ± 4.41**	**7.00 ± 4.40**	**≤0.05** ^a^
	**Chiasm D_max_ (Gy)**	**0.27 ± 0.17**	**0.35 ± 0.20**	**≤0.05**
	**Optic Nerve left D_max_ (Gy)**	**0.22 ± 0.20**	**0.31 ± 0.23**	**≤0.05**
	**Optic Nerve right D_max_ (Gy)**	**0.22 ± 0.17**	**0.28 ± 0.16**	**≤0.05**
	**Eye left D_max_ (Gy)**	**0.09 ± 0.07**	**0.13 ± 0.12**	**≤0.05**
	**Eye right D_max_ (Gy)**	**0.10 ± 0.06**	**0.15 ± 0.14**	**≤0.05**
	**Lens left D_max_ (Gy)**	**4 ± 2**	**7 ± 4**	**≤0.05** ^a^
	**Lens right D_max_ (Gy)**	**0.05 ± 0.03**	**0.07 ± 0.03**	**≤0.05**
	V98% (cm^3^)	0.1 ± 0.1	0.1 ± 0.1	n. s.
	V80% (cm^3^)	1.9 ± 1.6	1.9 ± 1.6	n. s.
	V60% (cm^3^)	4 ± 3	4 ± 3	n. s.^a^
	V40% (cm^3^)	8.6 ± 6.0	8.4 ± 6.1	n. s.
	V20% (cm^3^)	30.6 ± 22.9	29.1 ± 22.6	n. s.
	V10% (cm^3^)	112.7 ± 71.9	112.5 ± 81.2	n. s.

*Note*: Significant parameters are displayed in bold letters.

Abbreviation: n. s., not significant.

^a^ Parameters are normally distributed in usage of Shapiro‐Wilk test.

**TABLE 4:** Means and standard deviations of all parameters for lung treatment.

	Parameter	VOLO™	SEQU	*p*‐value
Lung	Number of nodes	47 ± 9	51 ± 11	n. s.^a^
	Number of beams	122 ± 35	145 ± 61	n. s.
	**Total MU**	**22 625 ± 9059**	**30 260 ± 12 585**	**≤0.05** ^a^
	**Treatment time (min)**	**37 ± 7**	**43 ± 14**	**≤0.05** ^a^
	CI	1.29 ± 0.17	1.28 ± 0.16	n. s.
	nCI	1.30 ± 0.17	1.29 ± 0.15	n. s.
	HI	1.54 ± 0.0	1.52 ± 0.07	n. s.
	GI	4.49 ± 0.99	4.31 ± 0.78	n. s.
	**Coverage (%)**	**99.6 ± 0.6**	**99.1 ± 2.2**	**≤0.05**
	D_min_ (Gy)	36.22 ± 2.91	35.49 ± 2.98	n. s.
	D_mean_ (Gy)	46.10 ± 0.83	46.24 ± 1.87	n. s.
	D_max_ (Gy)	38.90 ± 0.72	38.47 ± 1.37	n. s.
	D100% (Gy)	57.69 ± 0	57.69 ± 0	n. s.
	**D98% (Gy)**	**36.22 ± 2.91**	**35.18 ± 2.67**	**≤0.05**
	D2% (Gy)	55.53 ± 0.83	55.37 ± 0.93	n. s.
	Spinal Cord D_max_ (Gy)	1.55 ± 3.23	1.67 ± 3.32	n. s.
	Lung left D_max_ (Gy)	30.09 ± 25.21	29.65 ± 25.37	n. s.
	Lung right D_max_ (Gy)	38.56 ± 24.91	39.04 ± 24.30	n. s.
	Heart D_max_ (Gy)	7.77 ± 11.51	8.34 ± 11.10	n. s.
	Esophagus D_max_ (Gy)	6.85 ± 5.56	7.26 ± 6.02	n. s.
	V98% (cm^3^)	0.2 ± 0.1	0.1 ±0.1	n. s.
	V80% (cm^3^)	9.5 ± 9.3	9.1 ±8.7	n. s.
	V60% (cm^3^)	37.3 ± 43.2	35.2 ± 37.5	n. s.
	V40% (cm^3^)	93.8 ± 118.1	85.6 ± 100.8	n. s.
	V20% (cm^3^)	302.3 ± 370.3	286.8 ± 351.0	n. s.
	V10% (cm^3^)	803.9 ± 797.6	814.23 ± 841.0	n. s.

*Note*: Significant parameters are displayed in bold letters.

Abbreviation: n. s., not significant.

^a^ Parameters are normally distributed in usage of Shapiro‐Wilk test.

**TABLE 5:** Means and standard deviations of all parameters for liver treatment.

	Parameter	VOLO™	SEQU	*p*‐value
Liver	**Number of nodes**	**52 ± 11**	**57 ± 14**	**≤0.05** ^a^
	Number of beams	148 ± 42	171 ± 89	n. s.^a^
	**Total MU**	**23 400 ± 8027**	**30 296 ± 13 787**	**≤0.05** ^a^
	**Treatment time (min)**	**39 ± 9**	**46 ± 18**	**≤0.05** ^a^
	CI	1.25 ± 0.18	1.24 ± 0.15	n. s.
	nCI	1.25 ± 0.17	1.25 ± 0.15	n. s.
	HI	1.53 ± 0.05	1.54 ± 0.02	n. s.
	GI	3.93 ± 0.52	3.85 ± 0.55	n. s.
	Coverage (%)	99.5 ± 1.0	99.3 ± 1.0	n. s.
	**D_min_ (Gy)**	**36.33 ± 3.06**	**34.90 ± 3.70**	**≤0.05**
	**D_mean_ (Gy)**	**46.19 ± 1.25**	**45.74 ± 1.60**	**≤0.05**
	D_max_ (Gy)	57.32 ± 1.97	57.41 ± 1.94	n. s.
	**D100% (Gy)**	**36.66 ± 2.23**	**35.21 ± 3.41**	**≤0.05**
	**D98% (Gy)**	**39.07 ± 1.35**	**38.62 ± 1.59**	**≤0.05**
	D2% (Gy)	54.94 ± 1.92	54.71 ± 2.12	n. s.
	Spinal Cord D_max_ (Gy)	0.71 ± 0.58	0.75 ± 0.57	n. s.
	Kidney left D_max_ (Gy)	0.55 ± 0.43	0.59 ± 0.52	n. s.
	Kidney right D_max_ (Gy)	1.39 ± 2.13	1.30 ± 1.94	n. s.
	Liver 700 cm^3^ (Gy)	3.92 ± 3.67	4.09 ± 3.64	n. s.
	Stomach D_max_ (Gy)	5.07 ± 4.06	5.04 ± 3.82	n. s.^a^
	V98% (cm^3^)	0.2 ± 0.1	0.2 ± 0.1	n. s.
	**V80% (cm^3^)**	**17.5 ± 14.6**	**16.2 ± 13.9**	**≤0.05**
	V60% (cm^3^)	57.4 ± 50.4	57.1 ± 50.5	n. s.
	V40% (cm^3^)	128.8 ± 120.8	125.6 ± 121.7	n. s.
	V20% (cm^3^)	416.8 ± 439.2	405.2 ± 444.3	n. s.
	V10% (cm^3^)	1192.5 ± 1065.0	1208.1 ± 1105.8	n. s.

*Note*: Significant parameters are displayed in bold letters.

Abbreviation: n. s., not significant.

^a^ Parameters are normally distributed in usage of Shapiro‐Wilk test.

**FIGURE 2:**
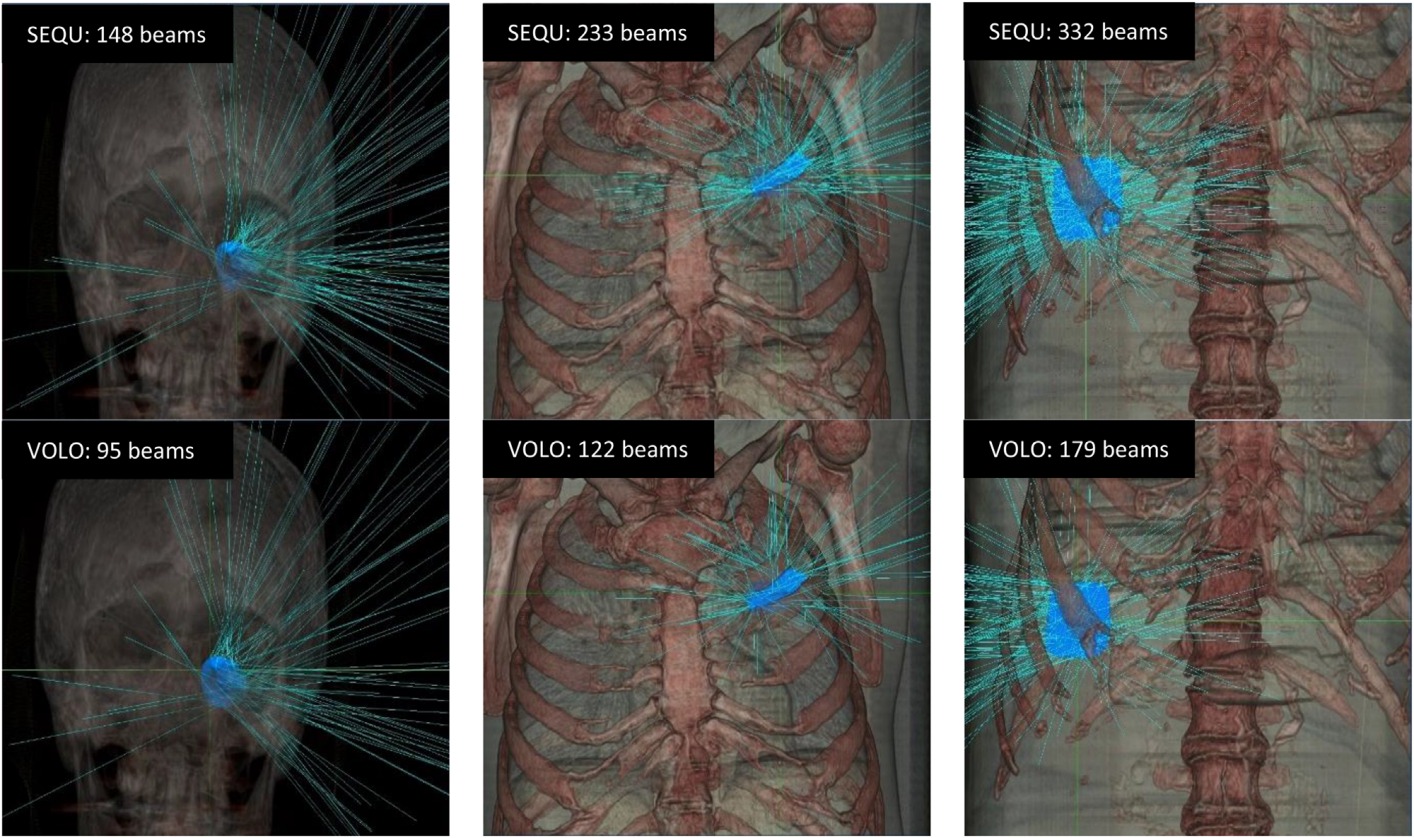
3D representation of the patient with the largest deviation in number of beams for the AN, lung, and liver metastasis groups. Blue is the PTV, and the thin light blue lines are the beams.

The largest difference in the number of beams between VOLO™ and SEQU for each entity is shown in Figure 2 (AN: 53 beams, lung metastases: 111, and liver metastases: 153).

### 3.2 AN treatment

AN treatments showed the most significant difference between VOLO™ and SEQU. A *p*‐value of ≤0.05 was determined for the number of nodes, number of beams, total MU, treatment time, and maximum dose to the left and right lens, left optic nerve and right eye. On average, VOLO™ optimization reduced the number of beams by 14.9%. D_max_ of brainstem is also significantly different with a *p*‐value of ≤0.05. Optimization with VOLO™ increases D100% by 0.38 Gy. For the OARs, all parameters are significantly different and on average VOLO™ results in lower dose values. In AN treatments, target volumes with VOLO™ optimization received 13.05 Gy, while with SEQU optimization, 12.67 Gy were deposited. The D98% is also significantly different. For VOLO™ plans, it is higher with 13.44 Gy compared to SEQU plans with 13.15 Gy.

### 3.3 Lung treatment

For lung treatments, only two normally distributed parameters have *p*‐values ≤0.05: the total MU, and the treatment time. The Wilcoxon signed‐rank test for paired samples showed a *p*‐value ≤ .05 for coverage and D100%. Optimization with VOLO™ increases D100% by 1.04 Gy and coverage by 0.5%. However, VOLO™ shows no consistent increase or decrease for the OARs in lung treatments. V10% is 10 cm^3^ smaller when VOLO™ is used. With VOLO™, the lung target volumes received an average of 0.73 Gy higher minimum doses than with SEQU.

### 3.4 Liver treatment

For liver treatments, significant differences are observed for the number of nodes, total MU, and treatment time (*p*‐value ≤ 0.05). Optimization with VOLO™ increases D100% by 1.47 Gy for the liver. The D98% is also significantly different here. VOLO™ treatment plans achieve a higher average D98% dose with 39.07 Gy compared to 38.62 Gy with SEQU. VOLO™ shows no consistent increase or decrease for the OARs. V10% is 16 cm^3^ smaller when VOLO™ is used.

### 3.5 Peripheral dose

**FIGURE 3:**
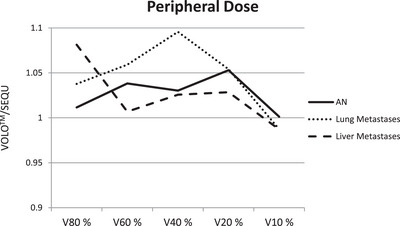
Peripheral dose as a ratio of VOLO™ and SEQU for AN, lung metastases and liver metastases.

In Figure 3 is the peripheral dose fall‐off shown for the three examined entities. Regarding the volume parameters V80% to V20%, the treatment plans optimized with SEQU are better. V10% for lung metastases and liver metastases is the only volume parameter where VOLO™ optimization minimally spares healthy tissue better.

### 3.6 Clinical evaluation

**FIGURE 4:**
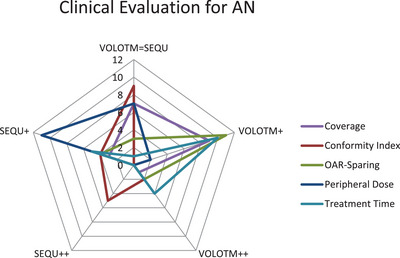
Clinical evaluation for AN treatment plans.

**FIGURE 5:**
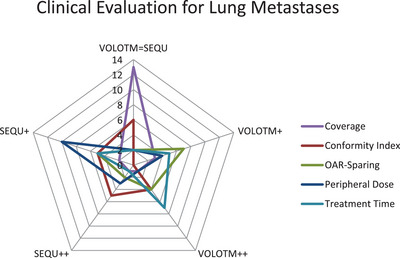
Clinical evaluation for lung metastases treatment plans.

**FIGURE 6:**
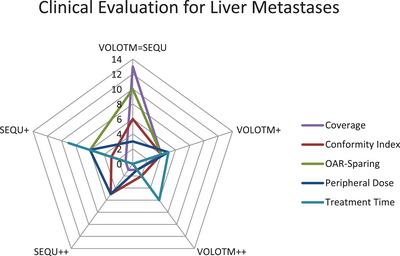
Clinical evaluation for liver metastases treatment plans.

The clinical evaluation of the three investigated entities (see Figures 4, 5, 6) shows no strong preference for one of the two optimization algorithms (SEQU++ or VOLO™++). In terms of AN treatment plans, VOLO™ tends to be better evaluated for coverage, OAR sparing, and treatment time. On the other hand, SEQU was preferred for peripheral doses. When evaluating the lung and liver metastases, the optimization algorithms showed more equality. For lung metastases, no optimization algorithm is preferred regarding coverage. However, a tendency toward SEQU was observed for peripheral doses. Both optimization algorithms tended to be evaluated equally in terms of coverage and OAR sparing.

## 4 DISCUSSION

The extent to which the optimization algorithms VOLO™ and SEQU differ from each other was investigated in this study using 60 clinical patients.

### 4.1 Reduction of plan parameters with VOLO™

In other study groups, VOLO™ has been shown to provide benefits through faster optimization time, increasing the efficiency of the treatment planning process and reducing the resulting treatment times.^6^, ^8^, ^9^ In the present study, an average reduction in treatment time of 14% was observed (AN: 12%, lung: 14%, liver: 15%). Calusi et al. concluded that VOLO™ achieved an average of 19% shorter treatment times.^8^ A reduction in treatment time using VOLO™ has advantages for patients with comorbidities, as shorter lying times lead to a more comfortable therapy and accurate application. For total MU, Calusi et al. observed an average reduction of 13% using VOLO™.^8^ The total average reduction in total MU in this study was 21%; 14% for AN, 25% for lung cases, and 23% for liver cases. Schüler et al. also observed significant reductions in total MU and treatment time in their study across all four of their planned categories (Brain Simple, Brain Complex, Spine Complex, and Prostate). The reduction in total MU ranged from 14% (Brain Simple Iris) to 52% (Brain Complex MLC) and the treatment time reduction ranged from 11% (Brain Simple Iris) to 22% (Brain Complex MLC).^9^ A reduction in total MU and better adaptability of the collimator to the target volume can reduce the peripheral dose in healthy tissue.

We observed that the number of beams and the number of nodes is, on average, less with VOLO™ than with SEQU. For the AN treatment plans, the number of beams was significantly reduced by an average 14% using VOLO™. The number of nodes decreased significantly by 15% for AN and 9% for liver metastases. The reduced number of beams and nodes may be linked to the slightly worsening target conformity for all examined entities with a mean CI of 1.31 for VOLO™, and 1.28 for SEQU for ANs. The mean CI for the lung metastases was 1.29 for VOLO™ and 1.28 for SEQU. For liver metastases, the mean CI was 1.25 for VOLO™ and 1.24 for SEQU. However, in the evaluation by Zeverino et al., the reduced number of beams did not worsen target conformity.^10^ As shown in Figure 1, there is a large difference in the number of beams between both optimization algorithms. Upon closer consideration, we observed that VOLO™ calculates with larger collimator sizes. For AN cases, 85% of the number of beams are applied via the larger of the two collimators; for lung and liver metastases, it is 87% and 80%, respectively. This could explain why the total MU and the number of beams decrease, but the CI increases minimally.

In the present paper, VOLO™ achieved better target coverage than SEQU for all three entities. The target coverage increased from an average of 99.04% to 99.65% for AN, from 99.06% to 99.61% for lung metastases, and from 99.27% to 99.52% for liver metastases. However, Calusi et al. and Zeverino et al. observed similar target coverage for both optimization algorithms.^8^, ^10^

The D_min_ and D_mean_ of the liver target volumes are also significantly different. Here, the average dose values are higher with VOLO™ than with SEQU and could lead to a better tumor control. This encourages the use of VOLO™ for liver cases, as the OAR exposure in the liver treatment plans did not show any significant differences between the two optimization algorithms.

The SEQU algorithm does not require weighting factors during treatment planning. In contrast, they can be used with VOLO™. For better comparability, the factor was set to one for the optimization with VOLO™. By increasing the weighting factor, the respective dose to the organs at risk can be spared more. If the tumor is close to the OARs, this may lead to a decrease in target coverage, and at a value <98 %, clinical application would no longer be acceptable in our department.

There are no significant differences between VOLO™ and SEQU in the dose exposure of OARs in liver and lung treatment plans. In contrast, the differences for all examined OARs for AN treatment plans were significant. The protection of the OARs is, on average, better with the VOLO™ optimization in the intracranial area, except for the brainstem. VOLO™ should still be preferred because the coverage and D_min_ were higher, as well as the sparing of all examined OARs. In 1 out of 20 lung metastases cases and in 2 out of 20 liver metastases cases, all DVH metrics were better with SEQU. For this reason, treatment planning should not be preferred with only one optimization algorithm. Indeed, it may be possible to achieve better results with the other optimization algorithm. Further comparative studies about other entities, for complex target volumes and target volumes adjacent to OARs with strict constraints are desirable, as some cases indicate SEQU generates better treatment plans than VOLO™.

### 4.2 Peripheral dose falloff

For V98%, V80%, V60%, V40%, V20%, and V10% VOLO™ treatment plans contained larger volumes on average compared to SEQU. Only V10% of the lung and liver treatment plans was smaller on average with VOLO™; 10.3 cm^3^ smaller for the lung, and 15.5 cm^3^ smaller for the liver. The study of Zeverino et al. observed higher dose values for the peripheral dose (V10% and V20%) in the VOLO™ treatment plans for lung than compared to their SEQU counterparts.^10^ However, higher values of V98% to V10% can be accepted because VOLO™ optimization improved the target coverage. A significant difference can only be seen in the liver V80% where VOLO™ was 17.48 cm^3^ and SEQU was 16.17 cm^3^. The dose falloff outside the target, which is described by the index GI, is steeper for SEQU for all three entities, opposite to the GI determined by Zeverino et al. We attribute this to the fact that SEQU calculates more with small collimators during the treatment planning process. For AN cases, 48% are applied via the smaller of the two collimators, compared to 43% and 50% for lung and liver metastases, respectively. This also explains why the number of beams in the SEQU treatment plans is always higher on average.

Despite the lower total MU and the lower number of beams, the examined VOLO™ treatment plans result in larger V98%–V20% volumes, exposing a greater volume of healthy tissue to the dose. However, increased D98% and D100% could potentially lead to a higher tumor control rate. As the V10% is reduced by 10.3 and 15.5 cm^3^ in the VOLO™ lung and liver treatment plans, respectively, compared to SEQU, we assume that VOLO™ shifts the dose in favor of the target volume.

### 4.3 Clinical evaluation

In the clinical evaluation, it was found that in AN treatment plans, VOLO™ was slightly preferred in three out of five evaluation criteria. In contrast, treatment plans for lung and liver metastases were scored more equally for both optimization algorithms. This indicates that the decision should be made on a patient‐by‐patient basis.

## 5 CONCLUSION

The results of this study indicate that VOLO™ should be used as a standard for AN cases instead of SEQU. Despite the lack of significance in the lung and liver cases, VOLO™ optimization is recommended because OAR sparing was similar, but coverage, D_min_, and D_mean_ were increased and thus better tumor control can be expected.

## AUTHOR CONTRIBUTIONS

Martin Thiele: Conceptualization (lead), Formal Analysis (lead), Investigation (lead), Methodology (lead), Validation (lead), Visualization (lead), Writing and Original Draft Preparation (lead), Writing and Review and Editing (lead). Kirsten Galonske: Investigation (supporting), Visualization (supporting), Writing and Original Draft Preparation (supporting), Writing and Review and Editing (supporting). Iris Ernst: Resources (lead), Formal Analysis (Supporting), Writing and Original Draft Preparation (supporting), Writing and Review and Editing (supporting).

## CONFLICT OF INTEREST STATEMENT

Martin Thile has nothing to disclose.
